# Genetic enhancement of phosphorus starvation tolerance through marker assisted introgression of *OsPSTOL1* gene in rice genotypes harbouring bacterial blight and blast resistance

**DOI:** 10.1371/journal.pone.0204144

**Published:** 2018-09-27

**Authors:** Kannan Chithrameenal, Ganesh Alagarasan, Muthurajan Raveendran, Sabariappan Robin, Suresh Meena, Ayyasamy Ramanathan, Jegadeesan Ramalingam

**Affiliations:** 1 Centre for Plant Molecular Biology and Biotechnology, Tamil Nadu Agricultural University, Coimbatore, India; 2 Department of Rice, Centre for Plant Breeding and Genetics, Tamil Nadu Agricultural University, Coimbatore, India; 3 Department of Soil Science and Agricultural Chemistry, Tamil Nadu Agricultural University, Coimbatore, India; National Institute of Plant Genome Research, INDIA

## Abstract

Phosphorus (P), an essential macronutrient, is a prerequisite for various plant-growth mechanisms including root establishment/development, early/late vegetative stage development and reproductive stage development. Rice (Oryza sativa) is very sensitive to P starvation. Most cultivated genotypes have poor tolerance levels to P deficiency and consequently the grain yield is severely affected by P starvation. Since P deficiency of soils is a major concern of rice production areas, it is necessary to develop new cultivars with enhanced P tolerance. This is also an expectation of farmers and the Agriculture ministry of southern states of India where rice cultivation is intensive. Our objective was to introgress the phosphorus starvation tolerance (*OsPSTOL1*) gene through marker-assisted backcross breeding (MABB) in to two intermediate genetic stocks of popular local-varieties namely, ASD 16 and ADT 43 which harbour bacterial blight and blast resistance (R) genes. To delve into the P starvation phenotypic effect, we have generated a set of four backcross inbred lines (BILs) with enhanced P starvation tolerance. The developed BILs showed altered root architecture pattern and greater root surface area with increased P uptake, confirming their adaptability to P deficient soil conditions. Further, a correlation between root traits and low/high P conditions indicates the function of introgressed *OsPSTOL1* in BILs. The enhanced root characteristics, therefore, enabled the plants to access and effectively absorb available nutrients from soil. In summary, the unique features of the *OsPSTOL1* BILs with bacterial blight and blast resistance can aid varietal development suitable for cultivation in P deficient soils.

## Introduction

Rice (*Oryza sativa* L.), as one of the major staple crops of the world, feeds billions of people. Increases in the global population demand an equitable rise in grain production. However, a recent report indicates that the current trends in crop yield increases will not meet the future demands. The reasons for low rice productivity are 1) decline in natural resources, including nutrients and water, 2) climate change, 3) poor harvest indices of existing crop varieties, 4) prevalence of pest and diseases, and 5) physical soil deterioration. Thus, sustaining the crop yields under changing climatic conditions has become the focus of major rice breeding programs. One of the most effective strategies is to pyramid multiple stress tolerances (both abiotic and biotic) and resource use efficiencies into elite genetic backgrounds through repeated backcrossing and molecular marker-assisted selection.

Among the abiotic and biotic stresses, bacterial blight and blast disease and P deficiency are of primary importance in southern India. One of the key nutrient sources for crop growth is phosphorus which is vital for root growth and for various key metabolic processes including synthesis of membranes and nucleic acids [1,2]. Low P availability is a major constraint for the production of rice in Asia, Africa, and South America [3]. Soil P deficiency is normally scattered, identifiable by sick patches across field plots.

Phosphorus as phosphate form, often binds to organic matter or chemicals in soils, which renders soil and fertilizer P unavailable to plants; it also reduces fertilizer use efficiency unless hydrolysis takes place to release this bound inorganic phosphate [4]. Increased fertilizer costs and gradual depletion of phosphate rock (source of P fertilizer), set a need to develop plant types with enhanced P uptake efficiencyto address P starvation [5].

The efficiency of soil P use in rice plant relates to the trait “phosphorus uptake 1” (*Pup1*), a major quantitative trait locus (QTL) on chromosome 12 [6]. *Pup1* was identified in a traditional “aus” rice genotype, Kasalath. Mapping studies have identified a protein kinase gene *OsPSTOL1* which promotes extensive root growth, enabling enhanced uptake of P from the soil [7–11]. Metabolic promotion of root system by a serine-threonine kinase *OsPSTOL1* in *Indica* rice genotype *Sahbhagi Dhan* has been reported [12]. The P_i_ starvation-induced (PSI) genes, *OsPSTOL1* and purple acid phosphatase (PAPs) genes induce overexpression of a transcription factor, *OsPHR2*, under low P conditions [13]. Most of the modern rice cultivars lack *Pup1* loci and thereby are highly susceptible to P starvation. Hence, introgression of *OsPSTOL1* into elite rice cultivars is one strategy towards developing high-yielding genotypes suitable for low P soils.

A further limitation to rice growth is the blast, a devastating rice disease caused by the fungus *Magnaporthe grisea*. Blast disease can lead to yield losses of up to 80% [14]. So growing blast resistant rice varieties would be the most cost-effective method for sustaining rice productivity in areas where blast is an endemic disease. So far, over 100 blast resistance genes have been mapped, and several major resistance genes (R-genes), including *Pib*, *Pita*, *Pid2*, *Pi9*, *Piz-t*, *Pi36*, and *Pi37*, have been cloned [15,16,17]. However, only a few genes exhibit broad-spectrum blast resistance [18]. Introduction of a major broad-spectrum R-gene, *Pi-k*^*h*^into a blast-susceptible *japonica* cultivar, Taipei 309, enhanced its tolerance against blast by deposition of callose in the host cells[18].

Another most serious disease in rice cultivation is bacterial blight (BB) [19], caused by *Xanthomonas oryzae* pv. *oryzae* [20,21]. BB infection develops at the maximum tillering stage and leads to blighting of the leaves and a consequent yield loss of up to 80% [22]. Chemical control is ineffective; so, once again, the development and introduction of resistant cultivars is the most economical, effective, and safest method of controlling this disease [23,24]. Over 40 bacterial blight resistance genes have been identified with Mendelian inheritance [25,26]. However, transferring a single resistance gene is not effective due to significant shifts in the virulence pattern of the pathogen population which led to breakdown of resistance. The most sustainable strategy is to pyramid multiple BB resistant genes into a single rice variety to prevent or delay the breakdown of resistance [23].

Two short-duration rice varieties, ADT43 and ASD16, developed by the Tamil Nadu Agricultural Unversity are very popular among farmers and consumers of southern India (www.agritech.tnau.ac.in/expert_system/paddy), but both are susceptible to both bacterial blight [27] and blast [28] and also P starvation intolerant. In addition, the soils of over 60% of the rice-growing areas in south India are P limited [29] which neccessitates an external application of high quantities of phosphorus to support the growth of phosphorus starvation intolerant varieties. This situation necessitates a combined approach for genetic enhancement of rice genotypes for tolerance to low soil P or enhanced P uptake/utilization efficiency and resistance to multiple diseases. Our previous attempts have led to the development of a near-isogenic line (NIL) of ADT43 (CB14002) introgressed with three BB resistance genes (*xa5*, *xa13* and *Xa21*) and a NIL of ASD16 (CB14004) pyramided with a blast resistance gene *Pi54* and 3 major BB resistance genes (*xa5*, *xa13* and *Xa21*) [27].

In the present study, our objective was to introgress and pyramid the major P starvation tolerance gene *OsPSTOL1* into these NILs, ADT43 (CB14002) and ASD16 (CB14004) which already harbor major disease resistance genes for blast and BB. However, this would involve the pyramiding of several loci/genes. So phenotypic selection might not be ideal or accurate to track multiple-loci. We,therefore, utilized marker-assisted backcross breeding (MABB) using gene-specific markers for foreground selection and polymorphic SSRs for background selection. Our purpose was to develop superior backcrossed rice progenies and to subject the identified progenies to comprehensive phenotyping to test traits related to P uptake efficiency and disease resistance.

## Materials and methods

### Plant materials

We used two NILs, ASD16 and ADT43 with bacterial blight and blast resistance and IR74-*Pup1* with *OsPSTOL1* as parent materials to develop multiple diseases resistant P starvation-tolerant rice genotypes. The standard marker-assisted backcross breeding (MABB) strategy [30–32] was employed to pyramid the genes. To characterize the root architectural adaptations to low P availability, plants were grown in three different environments including pot culture, hydroponics and gelrite medium.

**Fig 1:**
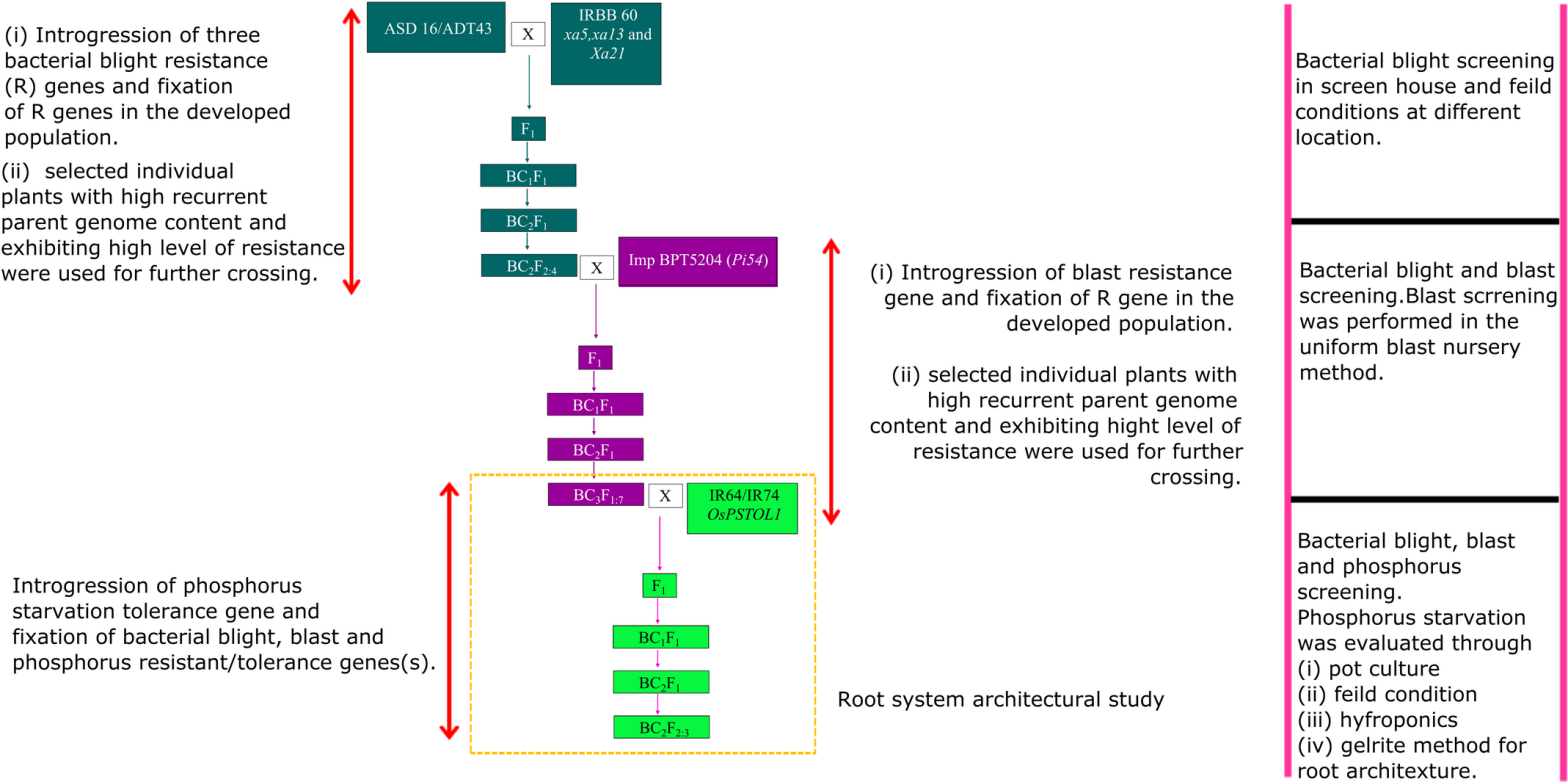
Hybridization plan for pyramiding ofbacterial blight, blastresistance and phosphorus starvation-tolerant gene (*OsPSTOL 1)*. Initially, the bacterial blight and blast resistance genes were pyramided independently in ASD16/ADT 43 vareities and named as CB14002/CB14004. The stabilized population harbouring bacterial blight and blast resistance genes were used as recurrent parent to introgress *OsPSTOL1* gene.

The crosses involved two near-isogenic lines namely CB 14002 and CB 14004 which were used as recurrent parents. CB 14002 isan advanced backcross (BC_3_F_7_) progeny of ADT43 (95.38% recovery of recurrent parent genome) having three BB resistance genes *xa5*, *xa13* and *Xa21*.CB14004 is an advanced backcross (BC_3_F_7_) progeny of ASD16 (96.71% recovery of ASD16 genome) with a blast resistance gene *Pi54* and threeBB resistance genes viz., *xa5*, *xa13* and *Xa21*[33–37]. CB14002 and CB14004 exhibited a high level of stable resistance to BB and blast under field conditions [38]. Donor parent comprises a set of NILs in the genetic background of IR64 and IR74 harboring *OsPSTOL1* loci from Kasalath (developed at IRRI, Philippines). Phenotypic evaluation of donor lines was carried out in the University field location(Paddy Breeding Station—TNAU, India, latitude: 11.003; longitude: 76.923; elevation: 419m). The results of the evaluation showed that IR74-*Pup1* displaying good agronomic traits, compared toother lines in terms of early maturity, semi-dwarf, high tillering and compact panicles without awns (data not shown). The scheme of hybridization programme is shown in Fig 1.

### DNA extraction and foreground selection

Genomic DNA fromyoung leaf sampleswas isolated by modified CTAB method [39]. For polymerase chain reaction (PCR), the isolated DNA wasdiluted with nuclease free water to a concentration of 40 ng/μl and stored at -20°C for further use. Gene specific markers were used to confirm target gene(s) in each backcross generation (Table 1). PCR amplification conditions maintained are as follows: *OsPSTOL1* (94°C for 5 min, followed by 35 cycles of 94°C for 30s, 58°C for 45 s, 30°C for 90 s and a final extension at 72°C for 10 min); *Pi54* (94°C for 5 min, followed by 35 cycles of 94°C for 1 min, 56°C for 1 min, 72°C for 1 min and a final extension of 72°C for 7 min); *xa13* (94°C for 5 min, followed by 35 cycles of 94°C for 1 min, 59°C for 1 min, 72°C for 90 s and a final extension of 72°C for 7 min); *Xa 21* (94°C for 5 min, followed by 35 cycles of 94°C for 45 s, 65°C for 1 min, 72°C for 90 s and 72°C for 7 min) and *xa5* (94°C for 5 min, 94°C for 1 min, 56°C for 1 min, 72°C for 90 s and 72°C for 7 min).To figure the *xa5* allelic pattern, 8 μl of PCR product was used for restriction digestion using BSRI enzyme at 65°C for 4h and separated on 1.5% agarose gel.

**Table 1:** Details on markers used in foreground selection of phosphorus starvation tolerance, blast resistance and bacterial blight resistance.

Targeted gene	Marker	Primer sequence (5’ - 3’)*	Enzyme	Chromosome	Reference
*OsPSTOL 1*	K 29-3F, 3R	F: TTCGTCCAGATGCTGCTATG R: TCTTCGGTGTAATTGGCACA	-	12	[9]
*OsPSTOL 1*	K 29-2F, 2R	F: CCCGTCTGCGTTCTACCTTA R: CTCCCGTCAAGCACAAATCT	-	12	[9]
*OsPSTOL 1*	K 29-1F, 1R	F: ATGGCCAACGGGGTAGAG R: GTCCAGGTAACCACGAGGAA	-	12	[9]
*OsPSTOL 1*	K 46–1	F: TGAGATAGCCGTCAAGATGCT R: AAGGACCACCATTCCATAGC		12	[9]
*Pi 54*	Pi 54 MAS	F: CAATCTCCAAAGTTTTCAGG R: GCTTCAATCACTGCTAGACC	-	11	Ramkumar et al. (2010) [37]
*xa 5*	xa 5_1F, xa 5_1R	F: CGGATAGCAGCATTTCCAAGAG R: GATTCCTTTAGCAAGGTGTG	BsrI	5	Iyer and McCouch (2007) [37]
*xa 13*	xa 13F, xa 13R	F: GAGCTCCAGCTCTCCAAATG R: GGCCATGGCTCAGTGTTTAT	-	8	Chu et al. (2006) [37]
*Xa 21*	Xa 21F, Xa 21R	F: ATAGCTAGTTCATAGAGG R: ACATCCGTCACTCTGCCA	-	11	Perumalsamy et al. (2010) [37]

*F- Forward primer, **R**- Reverse primer

### Background selection

Five hundred and twenty-three SSR markers, covering the entire length of all the 12 rice chromosomes were subjected to polymorphism survey of parental lines viz., CB14002, CB14004, and IR74-*Pup1*. We employed polymorphic marker-assisted background selection in BC_1_F_1_, BC_2_F_1_, and BC_2_F_2_ progenies to assess the recovery of the recurrent parent genome [40]. Additionally, we picturized R_P_G recovery using graphical genotypes (GGT) version 2.0 software [41].

### Phenotyping of phosphorus starvation tolerance under pot culture conditions

Test genotypes CB14002 IL16, CB14002 IL69, CB14004 IL4 and CB14004 IL52 and parents were raised under greenhouse conditions. The experiment was set with a series of pots containing prescribed dosage of nutrient fertilizers (150 N: 50 P: 50K kg /ha) and another series of pots with low P soil(nutrient mixture except Pto mimic low P condition). P was given as a single basal dose, while the N and K in 3 split doses [42,43]. Two replications were retained for each treatment under completely randomized design. Observations on P-related traits viz., root length (cm), root weight (g), root P content (mg/g dry weight of root), shoot P content (mg/g dry weight of shoot) and total P contents (mg/g dry weight of plant) were notedfrom five plants in each replication, at harvesting stage. P content was measured colorimetrically by vanadomolybdate yellow color method [44].

Also, we have evaluated the agronomical performance of improved and parental lines under P_def_ and P_suf_ at another location i.e. Agricultural college and research institute–Madurai, Tamil Nadu (9.925 Latitude and 78.119 Longitude) during summer’2018 (Mar’2018 to June’2018). The experiment was carried out in a 10 x 10m low P plot [less than 11 kg/ha of P; P_def_) and 10 x 10m normal soil [more than 11 kg/ha of P; P_suf_]. Crops were cultivated by adopting normal cultivation practices as mentioned in http://agritech.tnau.ac.in/sri.html.

### Evaluation for phosphorus starvation tolerance under hydroponic conditions

Yoshida nutrient solution [45] with 100% P and 50% P of phosphorus grade was formulated. Seeds of test genotypes (CB14002 IL16, CB14002 IL69, CB14004 IL4 and CB14004 IL52) along with parental controls were sprouted in moist paper towels and allowed to grow for ten days and later transferred to trays containing hydroponic solution. Two replications were maintained for each treatment under completely randomized design. The pH was maintained at 5.0, and the solution wasreplenished every two weeks. After 50 days of transplanting, full plants were collected individually and observations on root length (cm) and root biomass (g) were recorded. The plant roots and shoots were sampled to measure P content [44].

### Evaluation of root system architecture in the parents and backcrossed lines

Seeds were incubated at 37°C for 3 days to break the dormancy and sprouted in half strength MS media [46] with 0.3% gelrite containing two P levels (100% P and 50% P). Tubes were later placed inside a tissue-culture chamber for 14 days (4000 Lux, 25°C, and 16 h day/8h dark), and root zone wascaptured at a uniform angle, and magnification using a high-resolution digital camcorder. The parameters viz., average root width (cm), network depth (cm), maximum number of roots, network area (cm^2^) and network length (cm) were measured adopting the “GiA Roots” program[47].

### Evaluation of parents and backcrossed lines against blast and BB diseases

A uniform blast nursery (UBN) method was adopted for the artificial screening of parental lines and backcrossed lines for their responses to blast disease. Each entry was grown in a row of 70 cm long and 10 cm apart. A local susceptible variety CO39 was raised in between every five lines of BILs and parents. The entire nursery was bordered by a row of the susceptible variety CO39 and excessive nitrogen (100–120 kg N/ha) was given (½N as basal and ½ at 15 days after sowing) to all the entries. Blast pathogen isolates that were available at the Department of Plant Pathology, TNAU, Coimbatore, India were utilized for inoculation. Nursery beds were smeared with a mixture of blast spore suspension 1 x 10^5^ conidia/ml & 2% carboxymethyl cellulose (CMS) and plants were covered with polythene sheets during the night time. Scoring of test entries was performed through SES scale and readings on leaf blast severity in test entries were collected at 10-day intervals from 25 to 30 DAS [48,49].

For screening against BB pathogen, each test entry was raised with a spacing of 20 x 15 cm in two rows of 70 cm length. The nursery was encircled by 3 to 4 border rows of local susceptible variety ADT38. The resistant check variety IRBB60 was raised in between every five lines of BILs and parents. Adequate level of nitrogen (150 kg N/ha) was applied. Peptone sucrose agar was used to sub-culture the pathogen and 48-hour old pure slant culture was used to prepare cell suspension (10 ml sterile water per slant to get 10^8^ to 10^9^CFU/ml). Plants were inoculated atthe maximum tillering and booting stages [50]. Inoculations were done using scissors rinsedin bacterial suspension to cut the top 2 to 3 cm of leaves. Disease severity scoring was done after 15 days of inoculation visually by measuring the lesion size by following SES scale. Test entries with lesion length of < 5 cm were recorded as resistant and those with > 5 cm were scored as susceptible [51,52]. The homozygous BC_2_F_3_ lines, recipient parental lines (ADT43, ASD16) and local susceptible check ADT38 were evaluated along with the resistant control, IRBB60.

### Evaluation of agronomic performance and grain quality

In the BC_2_F_4_ generation, pyramided lines along with the parents were assessed for agronomic performance in a randomized complete block design with two replications with 20 x 15 cm plant spacing. The experiment was carried out at the Paddy breeding station of the TNAU, India;latitude: 11.003; longitude: 76.923; elevation: 419m). The data of five plants in each replication were recorded for various agronomic characters viz., days to 50% flowering (DFF), plant height (PH), number of productive tillers per plant (NPT), flag leaf length (FL), flag leaf breadth (FB), panicle length (PL), panicle breadth (PB), shoot biomass per plant (SB), number of filled grains per panicle (NFG), spikelet fertility (SF), 1000 grain weight (GW) and single plant yield (SPY) using the standard evaluation system of rice (IRRI 2002) [49]. The grain quality traits including hulling percentage, milling percentage, head rice recovery (HRR), kernel length before cooking (KLBC), kernel breadth before cooking (KBBC), length to breadth ratio (L/B), kernel length after cooking (KLAC), kernel breadth after cooking (KBAC), linear elongation ratio (LER), breadthwise expansion ratio (BER), volume expansion ratio (VER) and alkali spreading value (ASV) were determined [53].

### Statistical analysis

#### Breeding program

Dendrograms were built based on sequential agglomerative hierarchical nesting (SAHN) based unweight pair group method with arithmetic means (UPGMA) using numerical taxonomy and multivariate analysis system (NTSYS-PC) computer package [54], to ascertain genetic relationships and phylogeny. For assessment of agronomic performance and grain quality, data were subjected to ANOVA and Fisher’s LSD to compare the genotypic means. In addition, a phylogenetic tree was drawn based on agronomic data using the DARwin package (http://darwin.cirad.fr/).

#### Plant phenotyping studies

The P-related traits were analysed through Student’s t-test. Equality of variances and normality of variables were justifiedby *F*-test and Kolmogorov-Smirnov test, respectively.The gelrite growth data were subjected to single-data correlation analysis.

## Results

### Development of gene pyramided BILs of ADT43 and ASD16

Among the three *OsPSTOL1*-specific markers (K 29–1, K 29–2, and K 29–3), K 29–3 showed clear scorable polymorphism between the parents with unambiguous banding pattern when compared to K 29–1 and K 29–2 (S1 Fig). Thus, K 29–3 was used as foreground marker. Additionally, *OsPSTOL1* is verified through K46-1 marker analysis, to avoid recombination residues in *OsPSTOL1* region (S2 Fig). For background selection, polymorphism survey amongthe rice lines (IR74-*Pup1*, CB14002 and CB14004) was conducted using 523 genome-wide SSR markers. This studyidentified 81 polymorphic markers for CB14002/IR74-*Pup1*cross and 109 polymorphic markers for CB14004/IR74-*Pup1* cross (S1 Table and S2 Table). Homozygous lines for *OsPSTOL1*, blast and blight resistance genes with high recurrent parent genome contribution were found in BC_2_F_2_ generation (S3 Fig & S4 Fig). Homozygous plants for *OsPSTOL1* loci were later verified for blast (*Pi54*) and three (*xa13*, *Xa21*, and *xa5*) bacterial blight resistance genes using sequence tagged site (STS) markers (S5 Fig). A step-by-step procedure for BIL development and results obtained in each generation have been explained in the S1 Text.

### Evaluation of parents and backcrossed lines for phosphorus-starvation tolerance

**Fig 2:**
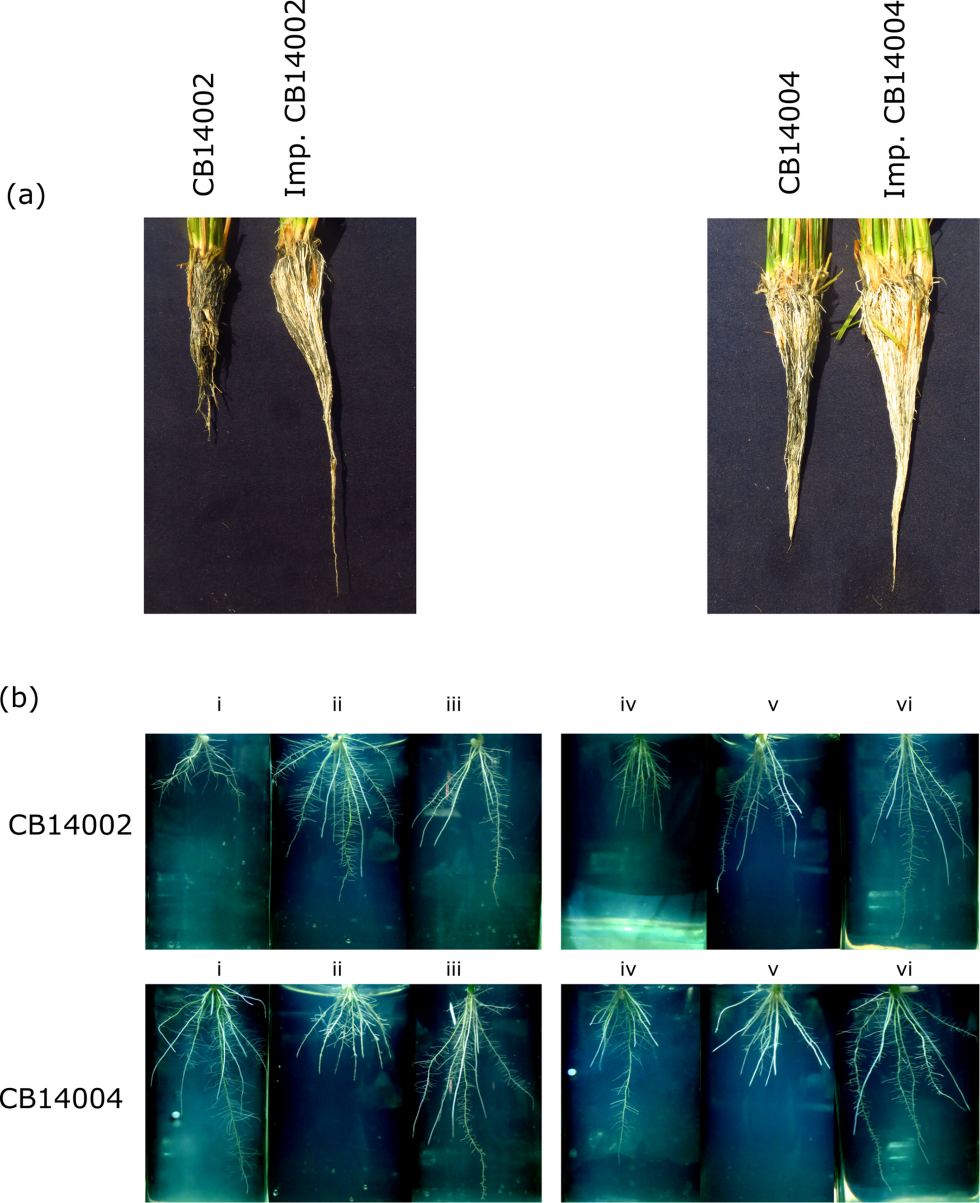
**(a)** Plants were grown under P deficient soil condition. The roots of improved and unimproved genotypes were shown in the image **(b)** Evaluation ofroot system architecture in BC_2_F_3_progenies under in-vitro conditions using MS+ gelrite medium. CB14002 genome background: i-iii = control; iv-vi = treatment; (i) CB14002(ii) CB14002 x IR74-*Pup1* (iii) IR74-*Pup1*(iv) CB14002 (v) CB14002 x IR74-*Pup1* (vi) IR74-*Pup1*. CB14004 genome background: i-iii = control; iv-vi = treatment;(i) CB14004 (ii) CB14004 x IR74-*Pup1* (iii) IR74-*Pup1* (iv) CB14004 (v) CB14004 x IR74-*Pup1* (vi) IR74-*Pup1*.Statisticalvalues were provided in Fig 4.

**Fig 3:**
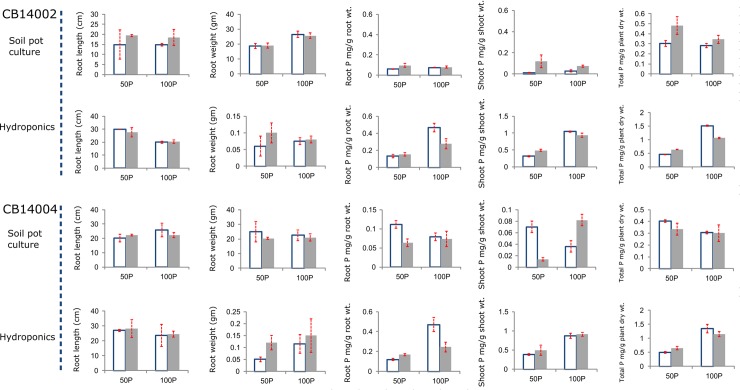
Comparison of important P-related traits of unimproved and improvedplants were shown. Shaded -improved lines; unshaded -unimproved. Mean of two replicates and standard deviations are shown. No significant statistical difference observed. Equality of variances and normality of variables were validated by *F*-test and Kolmogorov-Smirnov test, respectively).

Parental lines and progenies of four BC_2_F_3_ lines (two in CB14002 genetic background and two in CB14004 background) were tested at two P levels (0 and 100%). BC lines showed higher root length when compared to recurrent parents under low P conditions (Fig 2). In case of total P uptake, all the improved lines except CB 14004-IL4 had shown increased P uptake compared to their respective recurrent parents. But the root P uptake by all the four BILs was higher than the recipients, CB 14002 (0.06 mg/g) and CB 14004 (0.011 mg/g) under ‘0’ P applied condition (Fig 3).

Parental lines and *OsPSTOL1* introgressed lines were tested for their responses under two different levels of P (100% P and 50% P) in hydroponic conditions. CB14002IL69 showed higher root weight compared to its recurrent parent at moderate P stress (50% P). In case of total Puptake, CB14002IL69 outperformed the parents under low P condition (Fig 3).

The feild evaluation of improved lines having *OsPSTOL1* under low P revealed that grain yield of improved lines was significantly higher than recurrent parental lines (S1 Appendix). Recurrent parents showed nearly 12.5% reduction in yield under P-deficient conditions, whereas only a 2.5% yield reduction was noticed in ILs and donor lines under low P conditions.

### Effect of *OsPSTOL1* on root system architecture under normal and low P conditions

**Fig 4:**
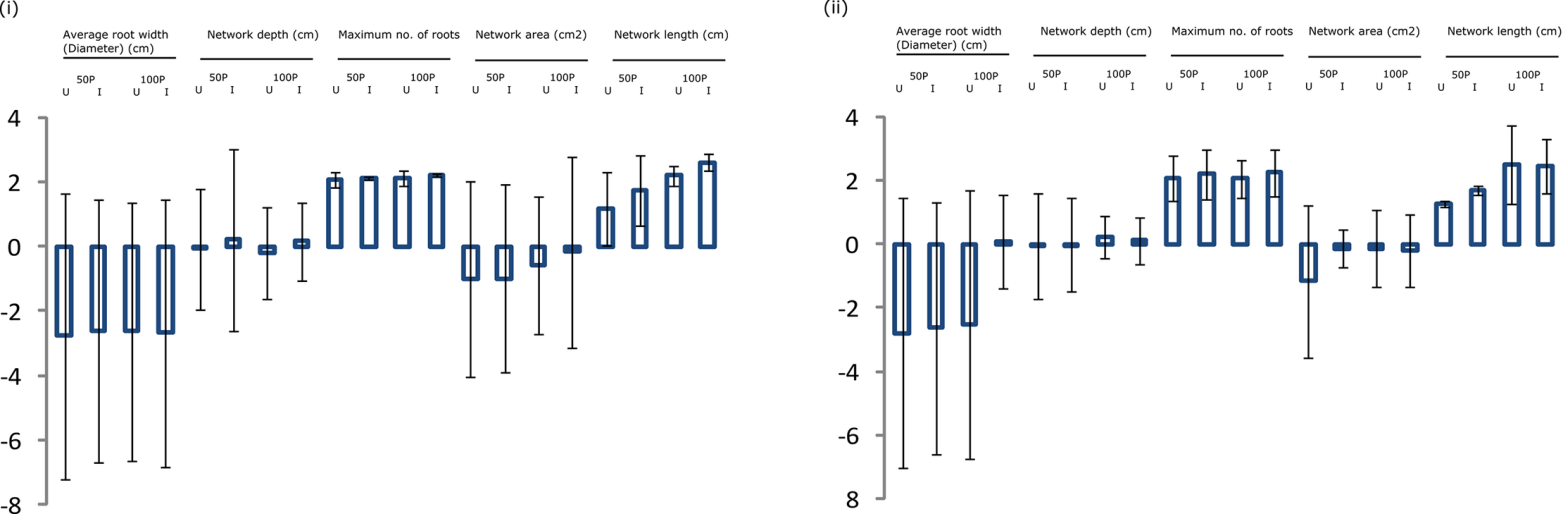
Statistical values of gelrite-based MS medium-grown plants were subjected for root system architecture evaluation. Mean values were Log transformed; SE- shown as bars.

Two BC progenies viz., CB14002 IL16 and CB14004 IL52 with acceptable agronomic and physiological performance in pot culture experiment were chosen and investigated for root architecture along with the donor and recipient parents through digital imaging. BC_2_F_3_ homozygous lines showed the maximum root number (132 to 144) compared to their respective recipient lines (118 to 119) under low P conditions (50% P). CB14004 IL52 showed higher network depth of 0.997 cm than the recipient parent (0.901 cm). Homozygous lines CB14002 IL16 (54.17 cm) and CB14004 IL52 (49.02 cm) showed increased network length when compared to their respective recipient parents under low phosphorus supply (Fig 4). Many of the P-related traits were at better correlation showing concurrent development in root system root system architecture (RSA)(Table 2).

**Table 2:** Pooled single data correlation matrix of root system architecture (RSA) of plants.

**Correlation matrix for the RSA data of plants grown in 50P**							
	a	b	c	d	e	f	g
b	-0.6610						
c	-0.8913	0.7621					
d	-0.8832	0.6479	0.9350				
e	-0.9112	0.7339	0.9733	0.9887			
f	0.5183	0.2115	-0.1282	-0.2239	-0.1919		
g	-0.3551	0.7816	0.5817	0.6293	0.6388	0.5129	
h	-0.6052	0.8769	0.8374	0.8074	0.8415	0.3471	0.9239
**Correlation matrix for the RSA data of plants grown in 100P**							
	a	b	c	d	e	f	g
b	-0.5647						
c	0.3686	-0.4976					
d	0.2572	0.2197	0.3656				
e	-0.3994	0.5010	0.2628	-0.0706			
f	-0.5517	0.9853	-0.6365	0.1326	0.3902		
g	-0.4846	0.9472	-0.7338	0.0553	0.2969	0.9871	
h	-0.2460	0.5943	-0.5331	-0.4841	0.5473	0.6377	0.6877

**Note:***a—Average root width (Diameter) (cm); b—Network depth (cm); c—Network length distribution; d—Maximum no*. *of roots; e—Network width (cm); f—Network area (cm2); g—Network surface area (cm2); h—Network length (cm)*.

### Evaluation of BILs for blast and bacterial blight resistance

**Fig 5:**
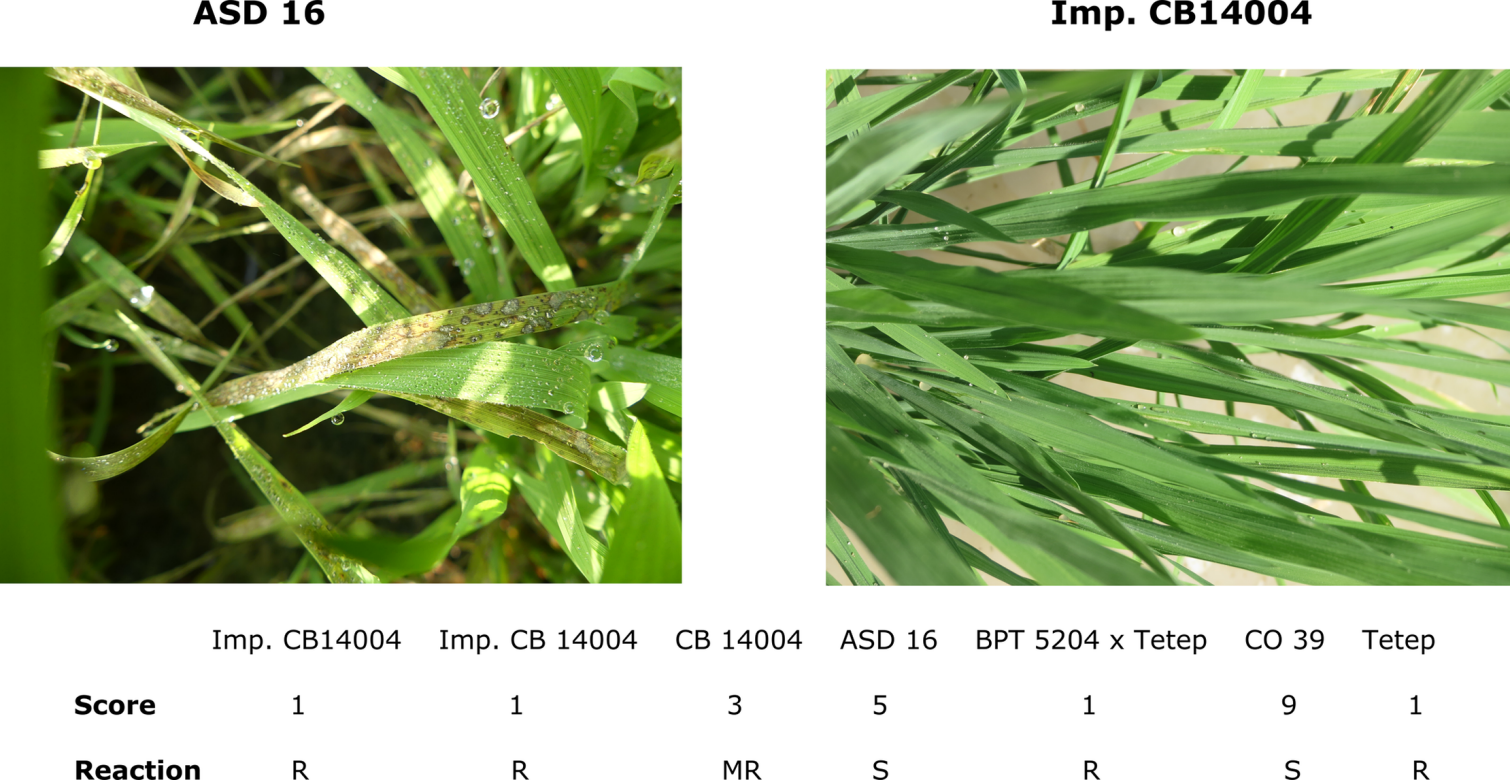
Blast resistance reaction of pyramided lines. Scoring of test entries was made through SES scale and readings on leaf blast severity in test entries at 10 days intervals from 25 to 30 DAS.

**Fig 6:**
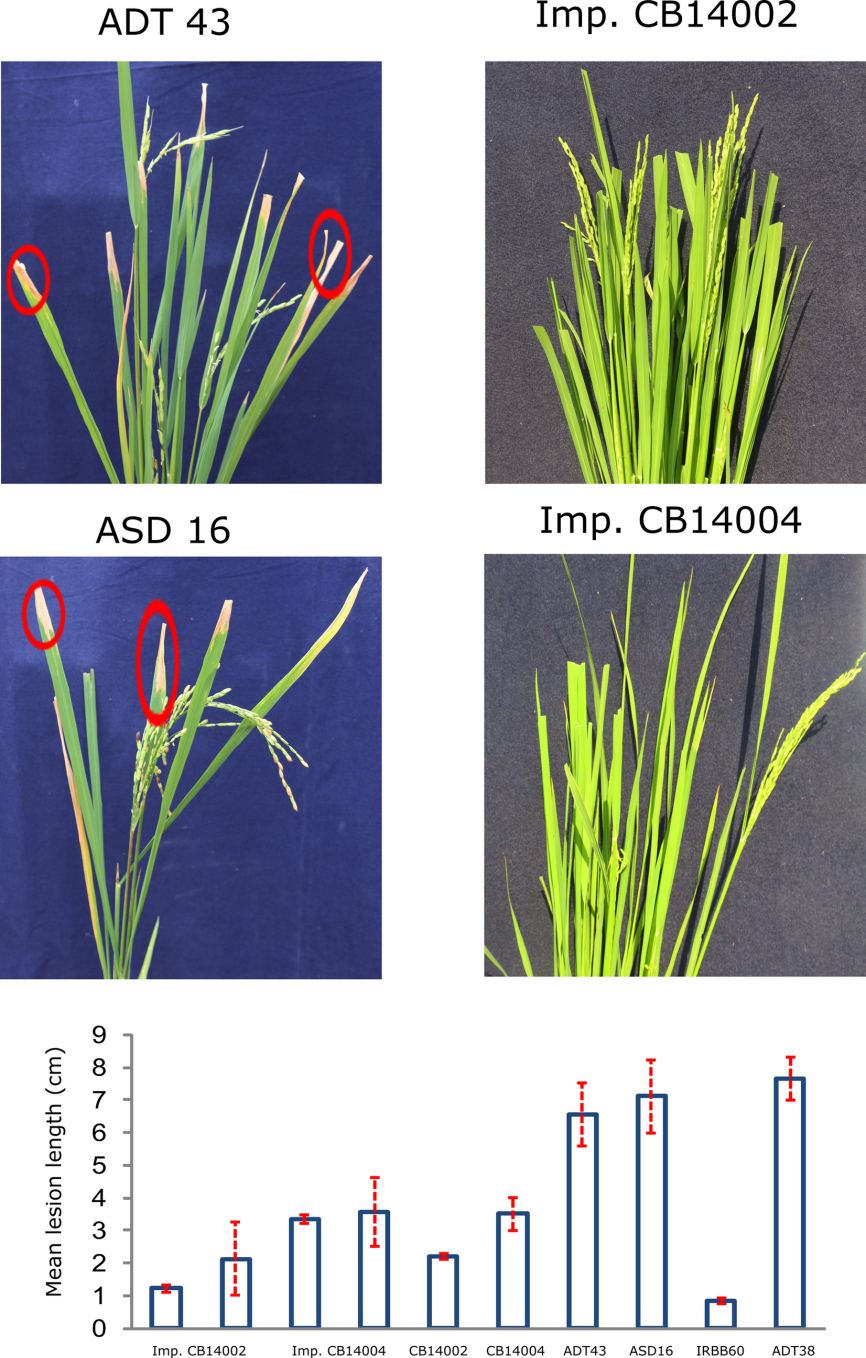
Bacterial blight resistance reaction of pyramided lines. Mean lesion length seen on 10 DAI. The graph shows mean value with calculated SD.

CB14004 IL4 and CB14004 IL52,retaining the broad-spectrum blast resistant gene *Pi54* showed high resistance to blast with a score of 1 (Fig 5). All the pyramided lines CB14002 IL16, CB14002 IL69, CB14004 IL4 and CB14004 IL52 harboring *xa13*, *xa5* and *Xa21* exhibited a high level of resistance to BB (lesion length of less than 3.5cm)(Fig 6).

### Cluster analysis

Two dendrograms (one for CB14002 genetic background and the other for CB14004 background) were generated based on the SSR data gathered in the background selection. Based on the dendrogram generated for CB14002 background, 5 genotypes, including the recurrent parent were grouped into two major clusters. Cluster I comprised CB14002 IL16 and CB14002 IL7 and cluster II comprised recurrent parent CB14002 and other pyramids CB14002 IL38, CB14002 IL69. In cluster II, CB14002 and CB14002 IL69 were adjacent to each othermaking a single line. In case of CB14004 background, there were two major clusters, cluster I and cluster II. Cluster I had CB14004 IL23 and cluster II posses CB14004 IL4, CB14004 IL52, and CB14004, in which CB14004 and CB14004 IL52 formed a single line (S1 Fig).

### Agronomic performance and grain quality characters of BILs

The agronomic performance of the advanced backcross-derived lines in BC_2_F_4_ generation was on par with the respective recurrent parent for most of the traits examined (Table 3). Two promising improved five-gene pyramids in CB14002 and two four-gene pyramids in CB14004 lines at BC_2_F_4_ generation, along with the donor and recipient parents were evaluated during the wet season of 2017 at TNAU, Coimbatore, India. The recipient parents, CB14002 and CB14004 recorded mean grain yield of 35.00 g/plant and 33.10 g/plant respectively, while the donor parent (*IR74 Pup1*) recorded 26.65 g/plant. The test entries *viz*., CB 14002 IL6 & 69 and CB14004 IL4 & 52 had higher grain yields than their respective recurrent parent. The improved lines showed nosignificant variation as compared to recurrent parent in terms of days for flowering, number of productive tillers, shoot biomass, and particularly for distinctness, uniformity and stability (DUS) characters. The genetic distance coefficient on twelve agro-morphologic traits of two pyramids and two parental lines revealed that all the pyramided lines were identical to the recipient parent. CB14002 and CB14004 were clubbed in a single cluster while in another cluster, only a solitary line, the donor parent *IR74 Pup1* was accommodated (S6 Fig). Most of the selections showed nearly similar performance to the recipient parents (CB 14002 and CB 14004) for grain quality traits. The line CB 14004 IL4 derived from CB 14004 showed increased kernel length than the recipient. In case of HRR (Head Rice Recovery), the improved lines performed more or less similar to their respective recipient parents. The two lines CB 14004 IL4 (8.5 mm) and CB 14004 IL52 (8.6 mm) reached higher KLAC values than the recipient CB 14004 (8.1 mm). CB 14002 derived lines showed higher ASV value (ASV 3) than the recipient CB 14002 (ASV 6) (Table 4).

**Table 3:** Agronomic performance of the pyramided lines in comparison to the recurrent and donor parent in BC_2_F_4_ generation.

Genotype	DFF	PH	NPT	FL	FB	PL	PB	SB	NFG		SF	GW	SPY
CB 14002 IL16	82.5bc	72.55c	24.0ab	35.10b	1.70c	29.59b	10.90ab	59.58a	113.5a	93.45a		18.15de	36.30ab
CB 14002 IL69	80.5c	75.25b	24.0bc	35.40a	1.38bc	26.80ab	10.65ab	61.67a	159.5e	96.13a		17.25a	35.80a
CB 14004 IL4	78.5b	84.10b	21.0ab	44.45b	1.60a	27.10ab	10.35ab	61.99a	104.5de	93.77a		26.75ab	38.65a
CB 14004 IL52	84.5bc	86.15d	23.5a	34.55b	1.92ab	27.04b	11.70ab	63.64b	107.0b	91.03a		25.85e	37.55ab
CB 14002 (recipient)	82.0c	71.24a	26.0c	32.85a	1.75abc	25.10b	10.80a	54.41a	137.5c	93.26a		16.35bc	35.00b
CB 14004 (recipient)	80.0a	91.15c	19.5d	45.05b	1.63abc	26.05a	12.55b	62.32c	123.5cd	89.89a		22.75cd	33.10c
IR74-*Pup1* (donor)	99.0d	77.35e	14.0e	36.82c	1.65d	29.95c	9.30c	46.97d	117.0f	92.88b		20.65f	26.65d
CD (0.05)	4.17	3.39	3.71	4.66	0.31	3.03	2.56	7.05	12.14	6.84		3.40	4.14

Note: statistical differences at *P*<0.05 are indicated with different letters.

DFF Days to 50% flowering, PH Plant height (in cm), NPT Number of productive tillers per plant, FL Flag leaf length (in cm), FB Flag leaf breadth (in cm), PL Panicle length (in cm), PB Panicle breadth (in cm), SB Shoot biomass (in g), NFG Number of filled grains per panicle, SF Spikelet fertility (in percentage), GW 1000 Grain weight (in g), SPY Single Plant Yield (in g).

**Table 4:** Grain and cooking quality attributes of improved lines.

Genotype	Hulling %	Milling %	HRR %	KLBC (mm)	KBBC (mm)	L/B	KLAC (mm)	KBAC (mm)	LER	BER	VER	ASV
CB 14002 IL16	71.19ab	53.97c	46.94c	5.5a	2.0a	2.75a	8.3a	2.7b	1.51	1.35a	3.2a	3b
CB 14002 IL69	72.24ab	60.24ab	50.86a	5.5a	2.0a	2.75a	8.5a	2.6ab	1.54	1.30a	3.1a	3a
CB 14004 IL4	73.98ab	64.02bc	48.66a	5.3a	2.4a	2.20a	8.5a	3.3ab	1.60	1.37a	3.3a	5a
CB 14004 IL52	74.83ab	62.52a	49.47b	5.1a	2.5a	2.04a	8.6a	3.1ab	1.68	1.24a	3.6a	5a
CB 14002 (recipient)	72.15a	65.17ab	54.04c	5.6a	2.0a	2.80a	8.7a	2.7a	1.55	1.35a	3.2a	6a
CB 14004 (recipient)	75.60b	62.89d	50.04d	5.1a	2.5a	2.04a	8.1a	3.6ab	1.59	1.44a	3.0a	5c
IR74-*Pup1* (donor)	70.84c	54.19e	41.68e	6.1b	2.1b	2.90b	9.5b	3.2c	1.55	1.52b	3.2b	1c
CD (0.05)	4.11	2.59	3.02	1.43	1.12	0.88	1.64	0.98	ns	0.42	1.48	1.14

Note: statistical differences at *P*<0.05 are indicated with different letters.

HRR Head Rice Recovery, KLBC Kernel length before cooking, KBBC Kernel breadth before cooking, L/B Length/Breadth ratio, KLAC Kernel length after cooking, KBAC Kernel breadth after cooking, LER Linear elongation ratio, BER Breadth wise expansion ratio, VER Volume expansion ratio, ASV Alkali spreading value (1–2 High and 6–7 Low).

## Discussion

### Gene pyramiding and fixation

Among various factors that limit rice productivity, bacterial and fungal diseases, and nutrient stress factors rank next to the environmental stresses. Severe disease prevalence and limitation of natural resources, including water, nutrients, etc., are posing the serious threat to rice productivity and hence genetic improvement of rice lines for disease resistance and resource use efficiency is one of the immediate long-term objectives in rice breeding. The gene *OsPSTOL1* showed to promote enhanced P uptake in rice [7–10]. In this study, a phosphorus starvation tolerance gene *OsPSTOL1*, a broad-spectrum blast resistance gene *Pi54* and 3 different BB resistance genes *xa5*, *xa13* and *Xa21* were introgressed and pyramided by following a carefully planned breeding programme. Marker-assisted backcrossing was followed up to BC_2_F_1_ and selfed to produce BC_2_F_3_. Selected back-cross progenies showed 86 to 89% of recurrent parent genome at BC_2_F_3_ generation. Graphical genotyping followed by construction of dendrogram using the genotyping data demonstrated that the selected backcross progenies are closer to their respective recurrent parents CB14002 and CB14004. High-level phosphorus uptake efficiency disease resistance to blast and BB were proved in the homozygous lines of BC_2_F_3_ through phenotypic screening. Similar investigations were published for marker-assisted gene pyramiding in different rice varieties for various stress conditions [40,51,52,55–60]. Stringent phenotypic selection was made in the BC_2_F_4_ generation for agronomic performance and for grain and cooking quality traits. All the selected improved lines tolerated low P condition by showing better performance than recipients and also with acceptable grain quality and yield traits.

### Phosphorus starvation tolerance

Phosphorus plays a vital part in the architecture and growth of the plant [61]. Higher grain yields under phosphorus deficiency can be accomplished by enhancing the P uptake from soil and intensifying the effective internal use of phosphorus in dry matter and the grain production. But very limited success have been reported so far [62]. The commercial, modern and new varieties show higher yields owing to the high harvest index. It was earlier hypothesised that greater increases in grain yield under phosphorus deficiency can occur by increasing the phosphorus uptake [63]. Hence, we planned to raise the phosphorus use efficiency (PUE) in crop plants to keep higher yield at low P soil [64]. Vegetative stage PUE should be improved in order to increase P uptake through any breeding approach. In soil-based screening experiments, P uptake varied between the genotypes, but the same results might not be echoed when genotypic diversity in PUE was examined [7,65]. This may be due to the difference in internal phosphorus use efficiency among the genotypes. Therefore, the current investigation was undertaken to increase the P uptake efficiency of few promising varieties of south India.

Evaluation of BC_2_F_3_ progenies, introgressed with *OsPSTOL1* gene under low P conditions demonstrated the significant differences between the parents and improved lines in terms of PUE. It is clear from the measure of root P content that all the four BILs exhibited enhanced P uptake efficiency on par with the donor under low P conditions when compared to the respective recipients. Eventhough the BIL CB14004-IL4 showed reduced total P uptake compared to the recipient CB 14004, the root P content was higher in the BILs. This proves the role of *OsPSTOL1* in enhancing the root morphology. Root system architecture and morphology can improve the P uptake under P deficient condition [66]. BC_2_F_3_ progenies introgressed with *OsPSTOL1* showed desirable root system architecture in gelrite media thus ensuring the lines’ ability to forage more water and nutrients from the soil under low P condition. BC progenies were found to have increased root network length and higher root number than the recipients under P starved condition. This observation was supported by the previous research finding by Wissuwa [7] in which it was stated that *OsPSTOL1* had positively influenced root morphology and root hair density which would strongly influence nutrient acquisition by plants.

In two identical screening experiments, and in field evaluation the improved lines showed better yield performance than recurrent cultivars. Field evaluation showed that an approximate of 10% yield is retained in improved lines under low P conditions. In our studies, improved lines performed physically better in low P conditions which demonstrates the field performance of the gene. A clear picture may be generated through further screening with higher sample size in developed rice lines. Although the high-throughput root phenotyping methods have the potential to explore the functions of an *OsPSTOL1* allele, selection of desired cultivars requires cross-validation with root parameters under field conditions.

### Disease resistance

Blast ranks first among the rice diseases affecting rice productivity in Asia because of its wide distribution and high incidence levels during growth seasons once conditions are favorable. It has been already demonstrated that pyramiding multiple blast resistant genes exhibiting resistance to multiple races/pathotypes is a powerful strategy to build broad and durable resistance in new cultivars [67]. The blast resistant gene *Pi-kh* (*Pi54*) confers a broad spectrum resistance for several races or isolates of *M*. *oryzae* [18]. In our bioassays, artificial screening of BC_2_F_3_ progenies revealed that all the BC progenies that were screened showed a high level of resistance which proved the efficacy of *Pi54* as reported earlier [40,68]. Bacterial blight is the second most devastating disease affecting rice cultivation in major rice-growing areas of Asia [22]. Several BB resistant genes have been mapped and a combination of three genes (*xa5*, *xa13*,and *Xa21*) is reported to be effective against bacterial blight isolates in major growing regions of the world [31,40,69]. Results of this study also endorsed the effectiveness of the combination of the above three genes against the Xoo isolates. In combination, these R genes are highly effective for atleast majority of races, if not all.

This study demonstrated the efficiency of marker-assisted backcross breeding combined with phenotyping in developing rice genotypes with improved disease resistance and enhanced level of tolerance to P starvation. These improved versions have to undergo a rigorous evaluation under field conditions for yield, disease resistance and tolerance for low P to assess its performance over locations in the subsequent years. Upon successful evaluation, these lines are expected toassist the farmers for cultivation in P limited soils.

## Conclusions

Strategies to develop cultivars with good nutrient use efficiency and durable resistance to pests and diseases are relying on the introgression of multiple genes into susceptible cultivars. Results of this study demonstrated accelerated development of rice genotypes upgraded for disease resistance and nutrient use/uptake efficiency through MABB. The fullest success of this work will provide a roadmap to rice breeders to undertake molecular breeding as a tool for developing resilient rice cultivars possessing disease resistance and P use efficiency. In the future, these lines will be forwarded to yield trials over multiple locations to study the stability in performance for the target traits. Furthermore, these backcrossed lines can be used as donor parents in breeding programmes, andcan also serve as a potential base material for studying host-pathogen interactions combined with P tolerance.

## Supporting information

S1 Appendix Total grain yield (kg/ha) of improved and parental lines under Pdef and Psuf conditions. (DOCX)

S1 Text Brief note on the development of BILs pyramided with OsPSTOL1. (DOCX)

S1 Fig Agarose gel electrophoresis pattern of gene based markers viz., (a) K 29–1, (b) K 29–2, (c) K 29–3 located within *OsPSTOL1* between the parents.
(TIF)

S2 Fig K46-1 marker-based confirmation of OsPSTOL1 gene in developed backcross progenies. (TIF)

S3 Fig **(A)**Graphical view of recovery of recurrent parent genome in BC_2_F_2_ lines of (i) CB 14002 X IR 74-*Pup1* (Plant # 16, #69, #38 and #7) and (ii) CB 14004 X IR 74-*Pup1* cross (Plant #52, #4 and #23). A, proportion of recurrent parent genome and H, proportion of heterozygous loci. **(B)** Genetic relatedness analysis of (i) CB14002 and, (ii) CB14004 derived lines through construction of dendrogram using SSR genotyping data.
(TIF)

S4 Fig Graphical view of recovery of recurrent parent genome in BC2F2 single plant with high RPGR. (A) CB 14002 X IR 74-*Pup1* (B) CB 14004 X IR 74-*Pup1*.
(TIF)

S5 Fig Foreground selection ofBC_2_F_2_ progenies using markers specific to (a) *OsPSTOL1* (K 29-3F, 3R); (b) *Pi54* (Pi 54 MAS); (c) *xa13* (using xa13F and xa13R); (d) *Xa21* (using Xa21F and Xa21R and (e) xa5 (using xa5_1F and xa5_1R). A, homozygous recurrent parent allele; H, heterozygous and B, homozygous donor allele respectively.
(TIF)

S6 Fig Neighbour-joining tree based on agro-morphological data. Numerical values were used to draw the tree in DARwin. (A high bootstrap value of 30k was made to get better results. Since the tree had a agronomically distant IR74 *Pup1* lines, we didnot includ outgroup in the analysis).
(TIF)

S1 Table List of polymorphic SSR markers used for background selection in CB 14002 X IR 74-*Pup1* cross. (PDF)

S2 Table List of polymorphic SSR markers used for background selection in the CB 14004 X IR 74-*Pup1* cross. (PDF)
