# Supplementary material for: Genetic enhancement of phosphorus starvation tolerance through marker assisted introgression of OsPSTOL1 gene in rice genotypes harbouring bacterial blight and blast resistance
Source: PLoS One. 2018 Sep 27;13(9):e0204144. doi: 10.1371/journal.pone.0204144 (PMC6159862; doi:10.1371/journal.pone.0204144)
Supplement: S1 Appendix — (DOCX) [file pone.0204144.s001.docx]

**Genetic enhancement of phosphorus starvation tolerance through marker assisted introgression of *OsPSTOL1* gene in rice genotypes harbouring bacterial blight and blast resistance**

**Kannan Chithrameenal^1^, Ganesh Alagarasan^1^, Muthurajan Raveendran^1^, Sabariappan Robin^2^, Suresh Meena^3^, Ayyasamy Ramanathan^2^ and Jegadeesan Ramalingam^1*^**

^1^Centre for Plant Molecular Biology and Biotechnology, Tamil Nadu Agricultural University, Coimbatore, India, ^2^Department of Rice, Centre for Plant Breeding and Genetics, Tamil Nadu Agricultural University, Coimbatore, India, ^3^Department of Soil Science and Agricultural Chemistry, Tamil Nadu Agricultural University, Coimbatore, India.

**Corresponding author information**

**Jegadeesan Ramalingam**

**Email id:** [**ramalingam.j@tnau.ac.in**](mailto:ramalingam.j@tnau.ac.in)

Note: the experiment is carried out in 10 x 10 m and extrapolated to yield kg/ha

S1 Appendix: The P improved lines, IL 16 and IL 69 (CB 14002 background) and IL 4 and IL 52 (CB 14004 background) were evaluated under P deficient (less than 11 kg/ha) and P sufficient (more than 11 kg/ha) were evaluated to assess their performance along with their respective parents and check IR 74 PUP lines in a replicated trial.
