# Supplementary material for: Genetic enhancement of phosphorus starvation tolerance through marker assisted introgression of OsPSTOL1 gene in rice genotypes harbouring bacterial blight and blast resistance
Source: PLoS One. 2018 Sep 27;13(9):e0204144. doi: 10.1371/journal.pone.0204144 (PMC6159862; doi:10.1371/journal.pone.0204144)
Supplement: S1 Text — (DOCX) [file pone.0204144.s002.docx]

**Genetic enhancement of phosphorus starvation tolerance through marker assisted introgression of *OsPSTOL1* gene in rice genotypes harbouring bacterial blight and blast resistance**

**Kannan Chithrameenal^1^, Ganesh Alagarasan^1^, Muthurajan Raveendran^1^, Sabariappan Robin^2^, Suresh Meena^3^, Ayyasamy Ramanathan^2^ and Jegadeesan Ramalingam^1*^**

^1^Centre for Plant Molecular Biology and Biotechnology, Tamil Nadu Agricultural University, Coimbatore, India, ^2^Department of Rice, Centre for Plant Breeding and Genetics, Tamil Nadu Agricultural University, Coimbatore, India, ^3^Department of Soil Science and Agricultural Chemistry, Tamil Nadu Agricultural University, Coimbatore, India.

**Corresponding author information**

**Jegadeesan Ramalingam**

**Email id:** [**ramalingam.j@tnau.ac.in**](mailto:ramalingam.j@tnau.ac.in)

**S1 Text:**

**Brief note on the development of BILs pyramided with OsPSTOL1**

Genotyping of F_1_s derived from CB14002/IR74-*Pup1* and CB14004/IR74-*Pup1* using K 29-3 has singled out six plants in CB14002/IR74-*Pup1* and sixteen plants in CB14004 /IR74-*Pup1* cross combinations, which were heterozygous for *OsPSTOL1* loci. In BC_1_F_1_, four plants were heterozygous in CB14002/IR74-*Pup1* cross combination and nine plants were heterozygous in CB14004/IR74-*Pup1* cross combination for *OsPSTOL1*. In the BC_1_F_1_, two plants viz., #2 (76.66%) and #9 (75.83%) from a cross of CB14002/IR74-*Pup1* had highest recurrent parent genome recovery of >75%. In the cross CB14004/IR74-*Pup1*, four plants #11 (75.81%), #13 (78.22%), #14 (76.61%), #28 (77.42%) and #29 (75.81%) with highest recipient parent genome recovery were chosen for generation advancement.

In BC_2_F_1_, five plants were heterozygous in the cross CB14002/IR74-*Pup1* and two in CB14004/ IR74-*Pup1*. Two plants #3 (87.5%) and # 22 (88.33%) with highest recipient parent genome recovery were taken in CB14002 x IR74-*Pup1* cross combination. Similarly, two plants viz., plant # 3 (86.29%) and #21 (88.71%) were chosen in CB14004 x IR74-*Pup1* and forwarded to BC_2_F_2_.  In BC_2_F_2_, 33 and 31 plants were homozygous for *OsPSTOL1* gene in the cross CB14002 x IR74-*Pup1* and CB14004 x IR74-*Pup1,* respectively. Four plants (#7, #16, #38, #69) in the cross CB14002/IR74-*Pup1* were classified as four -gene homozygous (*OsPSTOL1, xa5, xa13* and *Xa21*) and three plants (#4, #23, #52) in the cross CB14004/IR74-*Pup1* were found as five-gene homozygous (*OsPSTOL1, Pi54, xa5, xa13* and *Xa21*) in BC_2_F_2_. Two plants viz., #16 (86.6%) and #69 (88.33%) with maximum recovery of recurrent parent genome in the cross CB14002/IR74-*Pup1* were singled out and forwarded to BC_2_F_3_. In CB14004/IR74-*Pup1* cross, #4 (87.09%) and #52 (87.9%) plants were selected and selfed to produce BC_2_F_3_.
