# Supplementary figures and images for: Genetic enhancement of phosphorus starvation tolerance through marker assisted introgression of OsPSTOL1 gene in rice genotypes harbouring bacterial blight and blast resistance
Source: PLoS One. 2018 Sep 27;13(9):e0204144. doi: 10.1371/journal.pone.0204144 (PMC6159862; doi:10.1371/journal.pone.0204144)

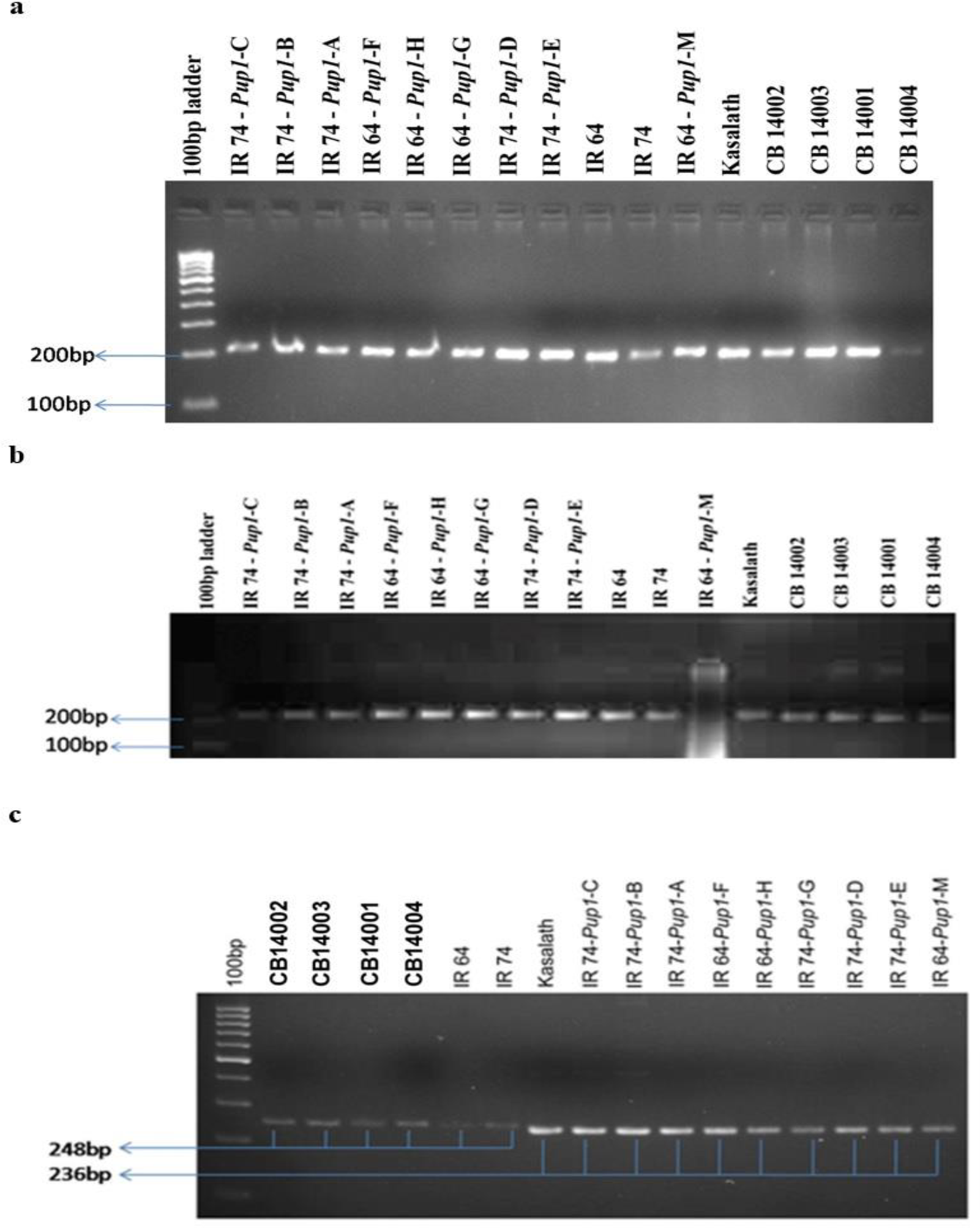

Supplement: S1 Fig — Agarose gel electrophoresis pattern of gene based markers viz., (a) K 29–1, (b) K 29–2, (c) K 29–3 located within OsPSTOL1 between the parents. (TIF) [file pone.0204144.s003.tif]

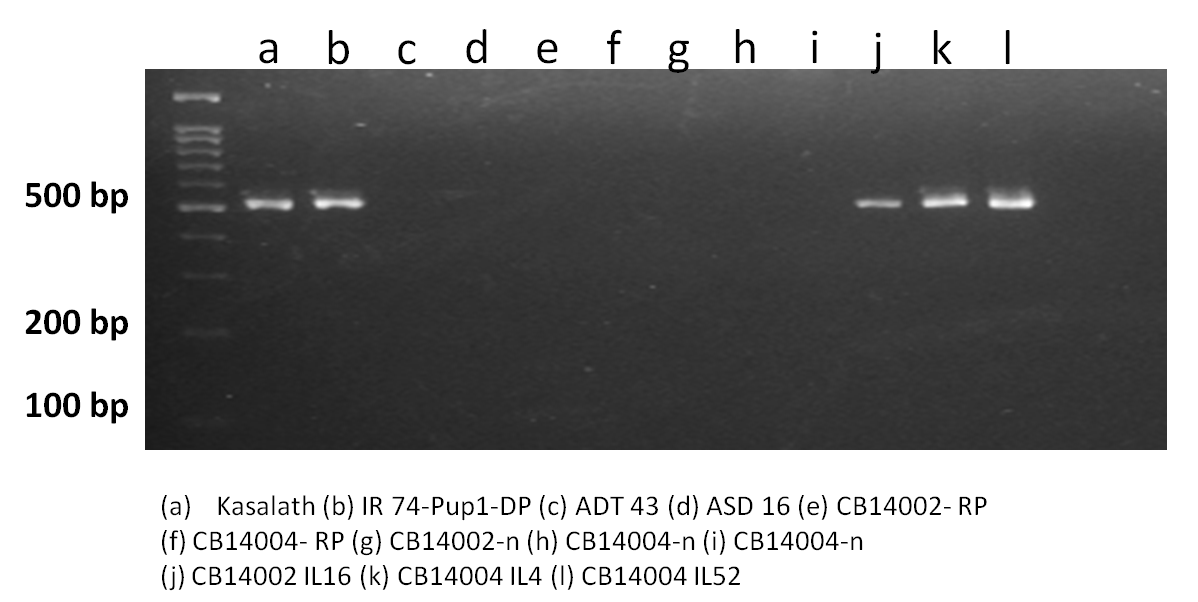

Supplement: S2 Fig — (TIF) [file pone.0204144.s004.tif]

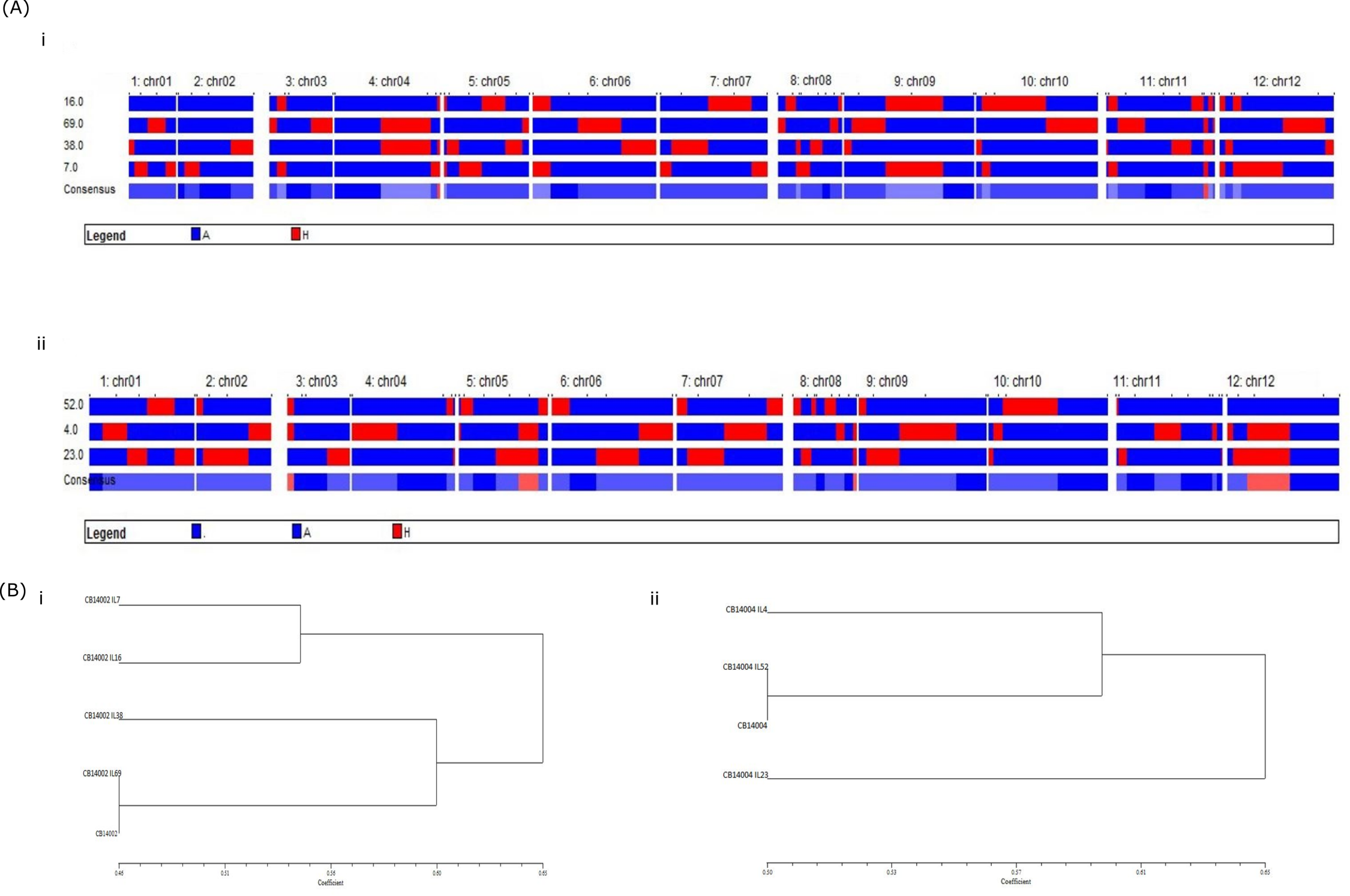

Supplement: S3 Fig — (A)Graphical view of recovery of recurrent parent genome in BC2F2 lines of (i) CB 14002 X IR 74-Pup1 (Plant # 16, #69, #38 and #7) and (ii) CB 14004 X IR 74-Pup1 cross (Plant #52, #4 and #23). A, proportion of recurrent parent genome and H, proportion of heterozygous loci. (B) Genetic relatedness analysis of (i) CB14002 and, (ii) CB14004 derived lines through construction of dendrogram using SSR genotyping data. (TIF) [file pone.0204144.s005.tif]

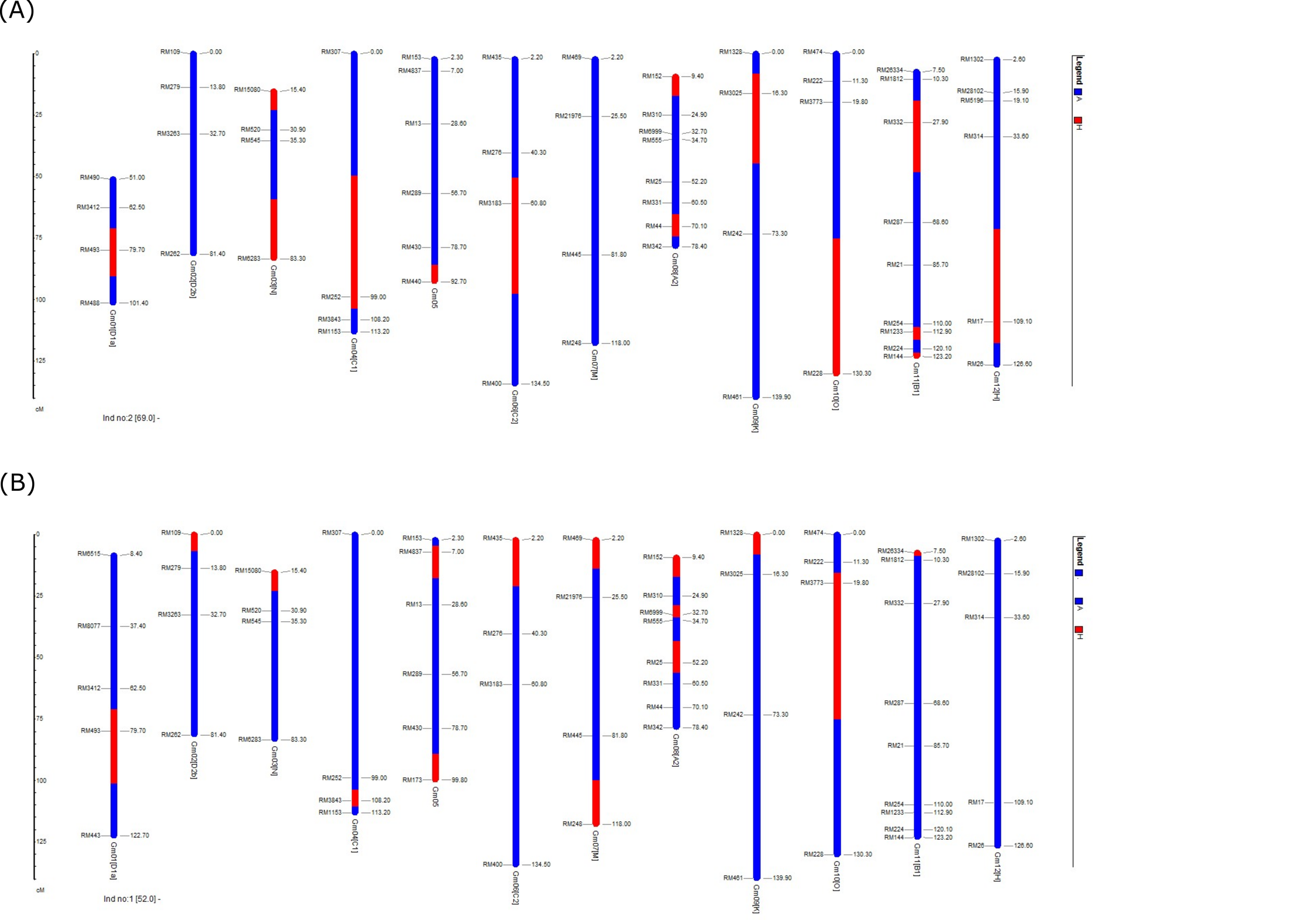

Supplement: S4 Fig — (A) CB 14002 X IR 74-Pup1 (B) CB 14004 X IR 74-Pup1. (TIF) [file pone.0204144.s006.tif]

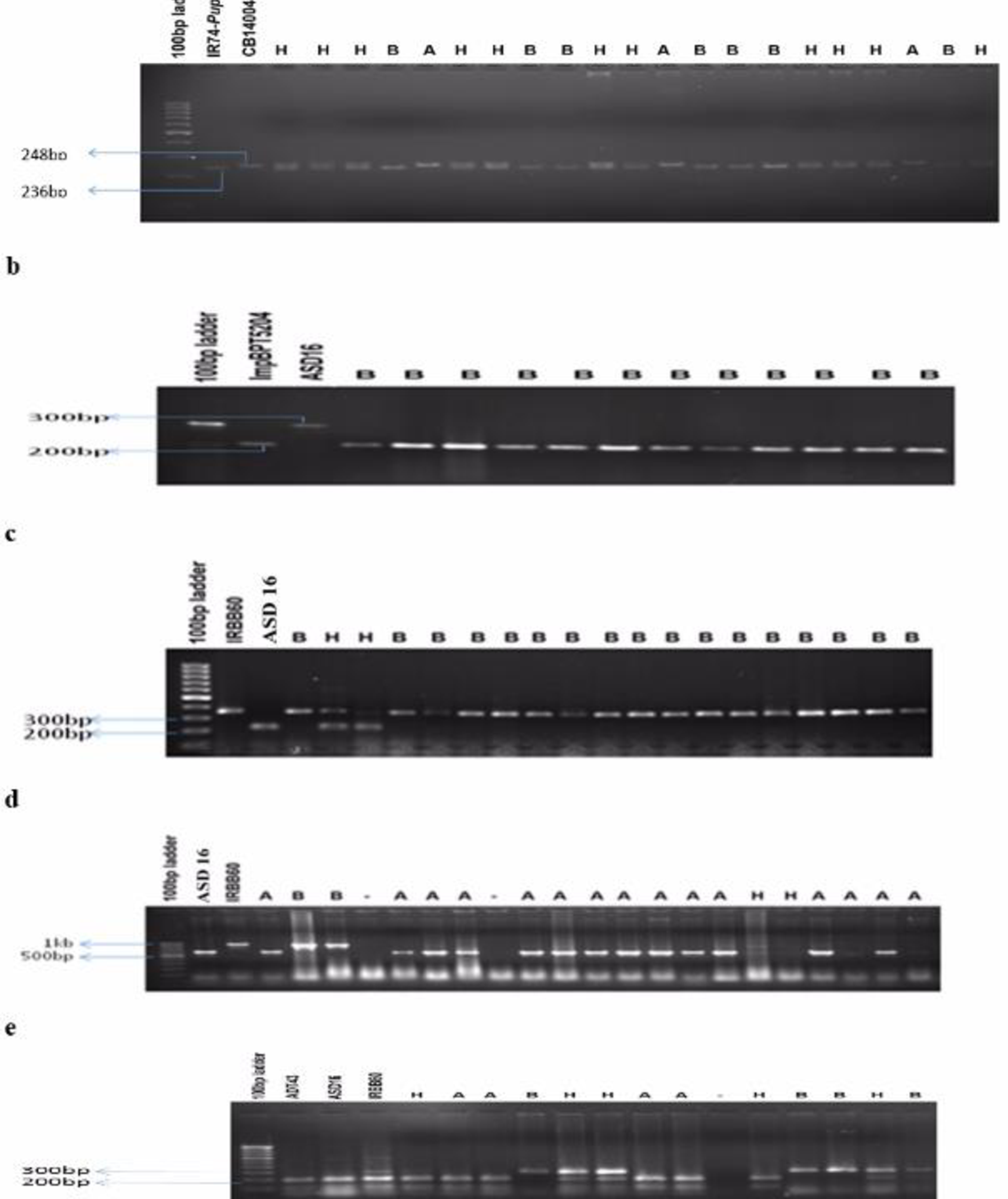

Supplement: S5 Fig — Foreground selection ofBC2F2 progenies using markers specific to (a) OsPSTOL1 (K 29-3F, 3R); (b) Pi54 (Pi 54 MAS); (c) xa13 (using xa13F and xa13R); (d) Xa21 (using Xa21F and Xa21R and (e) xa5 (using xa5_1F and xa5_1R). A, homozygous recurrent parent allele; H, heterozygous and B, homozygous donor allele respectively. (TIF) [file pone.0204144.s007.tif]

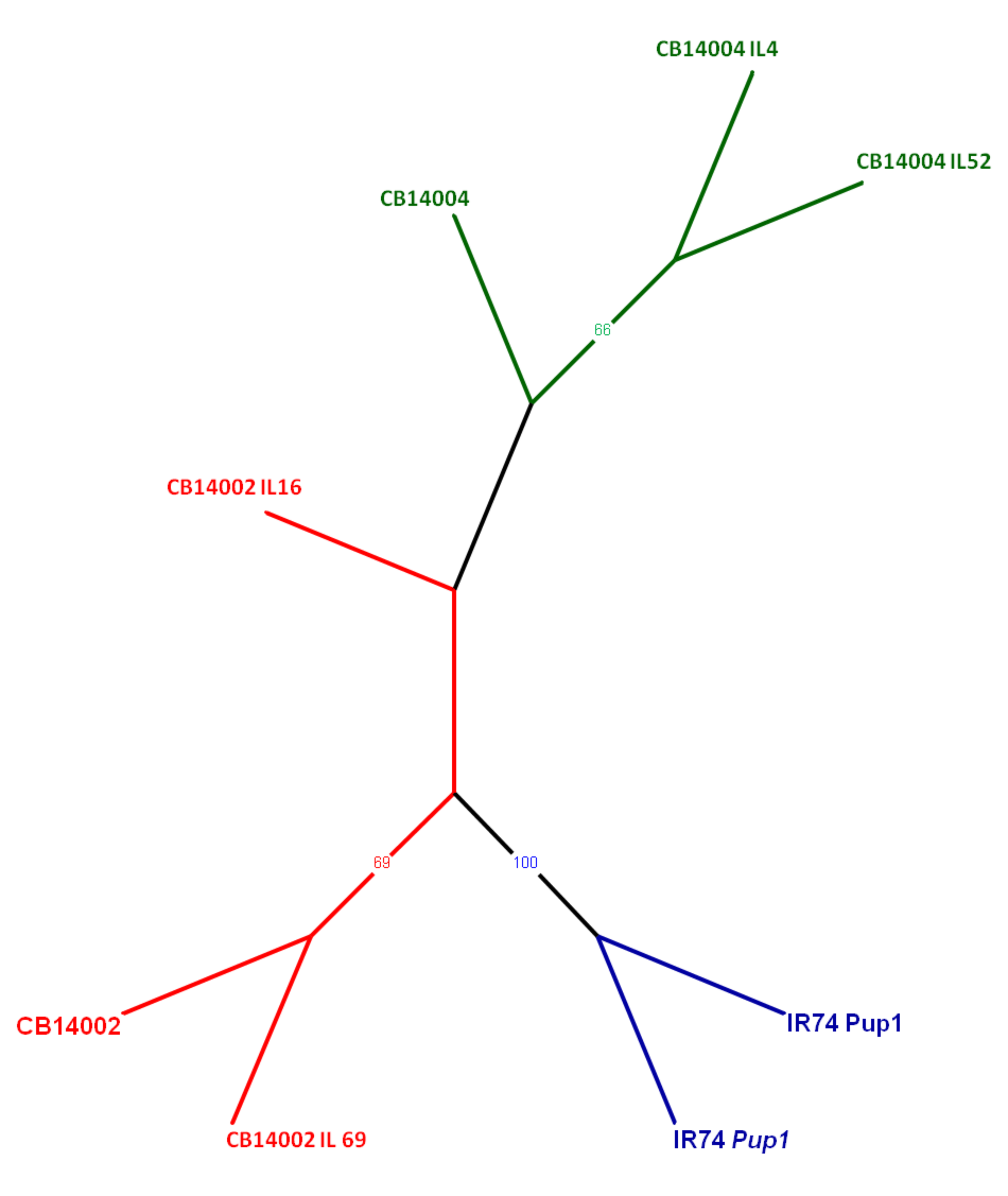

Supplement: S6 Fig — Numerical values were used to draw the tree in DARwin. (A high bootstrap value of 30k was made to get better results. Since the tree had a agronomically distant IR74 Pup1 lines, we didnot includ outgroup in the analysis). (TIF) [file pone.0204144.s008.tif]
