# Supplementary material for: Genetic enhancement of phosphorus starvation tolerance through marker assisted introgression of OsPSTOL1 gene in rice genotypes harbouring bacterial blight and blast resistance
Source: PLoS One. 2018 Sep 27;13(9):e0204144. doi: 10.1371/journal.pone.0204144 (PMC6159862; doi:10.1371/journal.pone.0204144)
Supplement: S1 Table — (PDF) [file pone.0204144.s009.PDF]

Table S1. Polymorphic SSR markers used for background selection in CB 14002 X IR 74-*Pup1* cross

Chromosome locus	Number of polymorphic markers got	Polymorphic markers selected (with interval of 10 cM or more between them)
1	5	4 (RM488, RM490, RM493, RM3412)
2	6	4 (RM3263, RM109, RM262, RM279)
3	5	4 (RM545, RM520, RM6283, RM15080)
4	5	4 (RM252, RM1153, RM307, RM3843)
5	5	5 (RM13, RM430, RM153, RM289, RM4837)
6	9	4 (RM400, RM435, RM3183, RM276)
7	8	4 (RM469, RM21976, RM445, RM248)
8	11	8 (RM310, RM555, RM25, RM331, RM342, RM44, RM6999, RM152)
9	9	4 (RM1328, RM242, RM3025, RM461)
10	4	4 (RM222, RM228, RM3773, RM474)
11	11	9 (RM287, RM26334, RM1812, RM332, RM21, RM254, RM1233, RM224, RM144)
12	9	6 (RM1302, RM28102, RM5196, RM314, RM17, RM26)
**Total**	**81**	**60**
