# Supplementary material for: Genetic enhancement of phosphorus starvation tolerance through marker assisted introgression of OsPSTOL1 gene in rice genotypes harbouring bacterial blight and blast resistance
Source: PLoS One. 2018 Sep 27;13(9):e0204144. doi: 10.1371/journal.pone.0204144 (PMC6159862; doi:10.1371/journal.pone.0204144)
Supplement: S2 Table — (PDF) [file pone.0204144.s010.PDF]

Table S2. Polymorphic SSR markers used for background selection in CB 14004 X IR 74-*Pup1* cross

Chromosome locus	Number of polymorphic markers got	Polymorphic markers selected (with interval of 10 cM or more between them)
1	10	5 (RM443, RM8077, RM473, RM3412, RM6515)
2	11	4 (RM3263, RM109, RM262, RM279)
3	5	4 (RM545, RM520, RM6283, RM15080)
4	5	4 (RM252, RM1153, RM307, RM3843)
5	16	6 (RM13, RM440, RM430, RM153, RM289, RM4837)
6	11	4 (RM400, RM435, RM3183, RM276)
7	8	4 (RM469, RM21976, RM445, RM248)
8	11	8 (RM310, RM555, RM25, RM331, RM342, RM44, RM6999, RM152)
9	11	4 (RM1328, RM242, RM3025, RM461)
10	5	4 (RM228, RM216, RM3773, RM474)
11	9	9 (RM287, RM26334, RM1812, RM332, RM21, RM254, RM1233, RM224, RM144)
12	7	6 (RM1302, RM28102, RM5196, RM314, RM17, RM26)
**Total**	**109**	**62**
